# The formation of episodic autobiographical memory is predicted by mental imagery, self-reference, and anticipated details

**DOI:** 10.3389/fpsyg.2024.1355343

**Published:** 2024-02-27

**Authors:** Diane Lenormand, Baptiste Fauvel, Pascale Piolino

**Affiliations:** Laboratoire Mémoire, Cerveau & Cognition (LMC2 UR 7536), Institut de Psychologie, Université Paris Cité, Paris, France

**Keywords:** episodic memory, SenseCam, consolidation, episodic autobiographical memory, sense of self, machine learning

## Abstract

**Introduction:**

Despite the ecological nature of episodic memory (EM) and the importance of consolidation in its functioning, studies tackling both subjects are still scarce. Therefore, the present study aims at establishing predictions of the future of newly encoded information in EM in an ecological paradigm.

**Methods:**

Participants recorded two personal events per day with a SenseCam portable camera, for 10 days, and characterized the events with different subjective scales (emotional valence and intensity, self-concept and self-relevance, perspective and anticipated details at a month, mental images…). They then performed a surprise free recall at 5 days and 1 month after encoding. Machine learning algorithms were used to predict the future of events (episodic or forgotten) in memory at 1 month.

**Results:**

The best algorithm showed an accuracy of 78%, suggesting that such a prediction is reliably possible. Variables that best differentiated between episodic and forgotten memories at 1 month were mental imagery, self-reference, and prospection (anticipated details) at encoding and the first free recall.

**Discussion:**

These results may establish the basis for the development of episodic autobiographical memory during daily experiences.

## 1 Introduction

Episodic memory (EM) is a long-term memory of everyday events, allowing to ground our sense of Self in a temporal continuity (Tulving, 2002). More specifically, EM allows to encode, store, and recall personally experienced events with their spatial and temporal context. Episodic memories are rich and detailed, as each of them associates several types of information: the target information (“what”), its external (“where” and “when”), and internal context (“how”), which includes the integration of sensory-motor information (e.g., visual, auditory, and tactile), phenomenological information (e.g., emotions, thoughts, self-reference), and idiosyncratic aspects of this event. Recollection of information in EM is characterized by an autonoetic state of consciousness where individuals mentally “travel” across time to reexperience a specific event (Tulving, 1985). Episodic memories of personal, real-life events are also called episodic autobiographical memories (EAM; Piolino et al., 2009; Staniloiu et al., 2020). However, autobiographical memory relates not only to episodic memories of events that have occurred during our lifetime but also to semantic memories (i.e., general personal events and facts about ourselves; Conway, 2009). Thus, EAM is a long-lasting EM mainly dependent on the Self, which processes new experiences and memories through the lens of personal relevance, emotions, attitudes, and goals (Conway, 2005). EM and EAM present some differences in their methodological approaches as the former is generally studied in laboratory settings, asking participants to recall lists of new material, and the latter examines the memory of specific events from one’s past (Roediger and Marsh, 2003). Previous studies with laboratory materials suggest that the emotional component (Yonelinas and Ritchey, 2015), self-relevance (Straube, 2012) and subsequent recalls or updates of information (Alberini, 2005; Dudai, 2012) are important determinants of whether some information will be captured in long-term EM, semantic memory, or forgotten.

Ecological and naturalistic paradigms are particularly well-suited for the study of EM, bringing it one step closer to the characteristics of EAM (Sonkusare et al., 2019). For instance, studies have used smartphones (Foudil et al., 2021), 360° videos (Serino and Repetto, 2018), and real-life photographs with a digital camera (Pathman et al., 2011). Another noteworthy example of this kind of paradigms uses wearable cameras to record features of real-life events, either during a specified walk (Jeunehomme and D'Argembeau, 2019; Khachatoorian et al., 2021) or during their day-to-day activities (Mair et al., 2017; Gelonch et al., 2020). Wearable cameras are small devices, often worn around the neck, that capture still images of the surrounding environment from the wearer’s perspective. Images can later be uploaded to a computer and be presented several times to amnesic participants to support the memory of new autobiographical events, by reviewing the pictures and taking memory tests a short time after encoding (Hodges et al., 2011), or as cues during retrieval (Chow and Rissman, 2017).

Despite some challenges regarding image quality, events’ sorting and qualifying as well as participant compliance and attention during recorded events, wearable cameras have already been successfully adapted to assess the nature of memories with the Remember/Know paradigm (Tulving (1985); see for instance Khachatoorian et al. (2021)). Furthermore, wearable cameras have already allowed to draw interesting conclusions regarding EM functioning. For example, Jeunehomme and D'Argembeau (2019) found that goal-directed events are two to three times less temporally compressed when mentally reexperienced compared to non-goal-directed events. St Jacques and Schacter (2013) studied the effect of an intermediate memory reactivation (48 h post encoding) on a subsequent memory test (48 h after memory reactivation). They found that memory performance is better when there is a higher level of matching between the retrieval cue and the encoding context. Wearable cameras could be particularly relevant to study long-term memory. For instance, studies have shown that, despite a high rate of forgotten events, an end-of-day review of the events improved memory performance at 1, 2, 3, and 8 weeks post intentional encoding (Finley et al., 2011; Mair et al., 2017, 2019). Milton et al. (2011) used a neuroimaging approach to assess recollection and familiarity at 36-h and 5-month retention intervals with pictures automatically taken by SenseCam. The medial temporal lobe (MTL; mostly the parahippocampal gyrus) activated only for familiarity-based retrievals at the 5-month delay, and neocortical regions such as the medial prefrontal cortex (mPFC) were more engaged for recollection-based retrievals. This is in line with the brain regions traditionally engaged in autobiographical memory (AM), which are for instance the mPFC (Markowitsch et al., 2003; Northoff et al., 2006; Brand and Markowitsch, 2008; Summerfield et al., 2009; Oddo et al., 2010; Martinelli et al., 2013b), in relationship with the involvement of the Self; the cuneus for mental imagery (Burgess et al., 2001; Piolino et al., 2009; Kim, 2018); and the hippocampus for the richness of specific events (Piolino et al., 2009; Moscovitch et al., 2016). More specifically, in a recent review, Daviddi et al. (2023) identified a differential temporal activation of regions within the default-mode network for EAM depending on the early phase (construction) versus late phase (elaboration) of the retrieval mode (see for instance Holland et al. (2011), McCormick et al. (2018), Audrain et al. (2022)). The involvement of these regions in the retrieval of episodic autobiographical memories can thus be differentiated between the two phases. On the one hand, along the temporo-parietal axis, the left hippocampus and the posterior cingulated cortex are activated during both construction and elaboration; however, construction selectively recruits the right hippocampus, the ventromedial PFC, the precuneus, and regions in the frontal lobe, while elaboration focuses on the inferior frontal gyrus. In line with these findings, the recruitment of the mPFC observed for the SenseCam pictures during long-term recollection-based retrievals (Milton et al., 2011) suggests that SenseCam paradigms would specifically enhance construction-based processes. Thus, it seems possible to resort to paradigms that use wearable cameras to study the formation of episodic autobiographical-like memories (Dubourg et al., 2016).

The present study aimed at predicting how personal, real-life events would be stored in long-term memory (i.e., remembered or forgotten) at short (5 days) and long (1 month) delays. The participants were asked to record two personal events a day with a wearable camera for 10 days. They characterized each event using different subjective scales (emotion, self-relevance, estimated memorability…). Two surprise free recall tests were done 5 days and 1 month after encoding, respectively. The second free recall was followed by a cued recall based on photos of the personal recordings. Based on previous studies, we expected a decline in memory recall over time and a strong substantial role for events’ self-related processes, emotional intensity, and valence in predicting the status of subsequent memories (forgotten or episodic) at a long delay.

## 2 Materials and methods

### 2.1 Participants

Twenty-five participants were recruited for the present study (recruitment period: January 1, 2018 to May 31, 2019). This number was based on previous studies on long-term memory using wearable cameras [e.g., Finley et al. (2011); Milton et al. (2011)]. Three older participants were excluded because they scored too low on the Mini-Mental State Evaluation (MMSE; ≤28; Folstein et al., 1975) or on the Geriatric Depression Scale (GDS; ≥10; Yesavage et al., 1982). Therefore, a total of 22 participants aged between 18 and 65 years old (M = 39.6, SD = 16.2, 59% female) took part in the study, each taking two pictures a day for 10 days, for a total maximum of 440 events. All the participants had to be free of physical diseases, neurological and psychiatric issues and be active and autonomous. The choice of this age group was motivated by plans to apply such an experimental paradigm in an aging study, that is, with different age groups. The participants provided written informed consent and freely took part in the study. The study received approval from the Paris Institute of Psychology local committee.

### 2.2 Material and procedure

**Figure 1:**
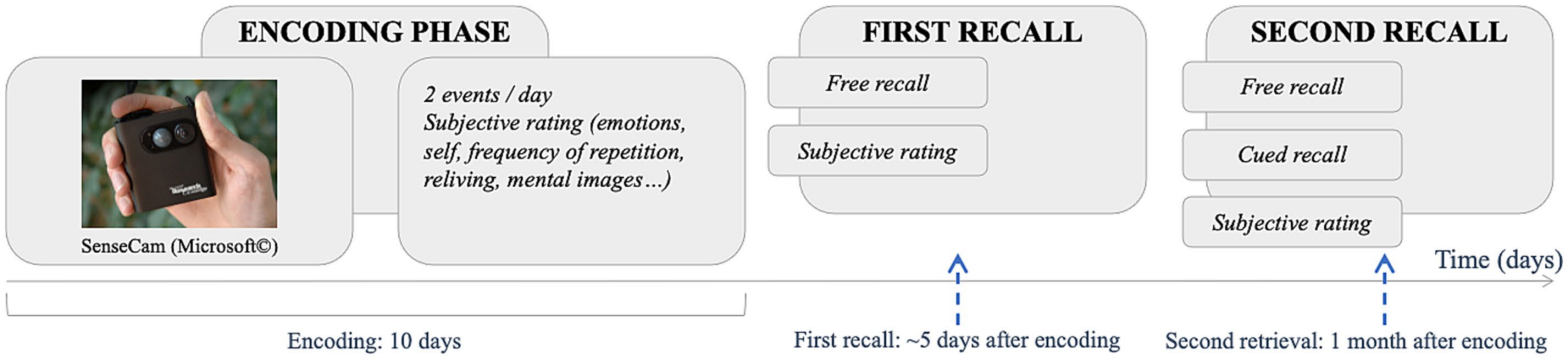
Procedure. During the encoding phase, the participants recorded two events a day for 10 days using a SenseCam wearable camera, that they rated with provided scales. They rated them again at the end of the first free recall, and after the second retrieval (free and cued recalls).

The experiment consisted of three main phases (Figure 1). During the first phase (i.e., the encoding phase), the participants recorded and assessed daily events using a wearable camera SenseCam. The second and third phases consisted of memory recall tasks at 5 days and one-month intervals after the encoding phase. Participants were informed that the research intended to study daily life events, but no mention was made of subsequent memory retrieval. More specifically, the participants were told that for the second phase they would “talk [with the experimenter] about how the 10 days went, and make sure everything went smoothly” (translated from French), and for the third phase, they would come back to the lab for some final neuropsychological tests (control tests).

#### 2.2.1 Encoding phase

Each participant was given one SenseCam wearable camera, alongside the experiment notebook, general guidelines and functioning instructions. SenseCam, developed by Microsoft Research, is a wearable camera with a wide-angle (fisheye) lens, ensuring a large point of view that encompasses everything the user sees. The user wears the camera around their neck, on a cord, for a first-person perspective. The pictures taken by SenseCam cameras are at VGA resolution (640×480 pixels) and are stored as .jpg on internal memory before being exported to a computer. SenseCam typically takes one picture every 30 s.

Participants had to select and record two events per day for 10 days by turning on the camera when a specific event happened, from its beginning to its end. More specifically, the participants were asked to “wear the SenseCam camera for 10 days to record two SPECIFIC events per day in your everyday life. Every day, you will choose TWO moments during which you think you will live something salient” (translated from French). Therefore, each participant had 20 memories encoded at the end of the encoding phase, providing a total of 440 memories (20 events x 22 participants).

The events had to be specific, that is, they had to target some significant activities and personal goals in everyday life. For instance, a walk in a park, cooking a recipe, a subway ride, grocery shopping, taking measures for new pieces of furniture, a family dinner, a work meeting, a visit to a museum, etc. would be considered as personal specific real-life events. At the end of each day, events were given short titles by the participants and assessed in the experiment notebook using 11 Likert scales ranging from 0 (‘no, not at all’) to 5 (‘yes, very much’) to identify the nature of the event and different aspects of recollective experience. These scales (listed in Table 1) were inspired by previous studies on EAM covering different retention delays [e.g., Piolino et al. (2004); Viard et al. (2007)]. They consisted in emotional intensity, emotional valence, self-relevance, self-concept, perspective, thinking, conversation, anticipated details, remembering, reliving, mental images. The encoding was incidental, as the subjects did not know that they would take two memory tests nor that the SenseCam images (that they could not see again) would be used for that purpose after a delay of 1 month.

**Table 1:** List and description of the variables.

Variable	Description
Emotional intensity	Intensity: emotional intensity
Emotional valence	Valence: emotional valence, whether the event is pleasant or unpleasant
Self-relevance	Personal importance of the event, whether the event is significant
Remembering	Knowing or remembering, whether one knows the event happened or actively recollects it
Perspective	Observer or actor perspective, whether one observes the images in the memory or whether one sees them as they lived the event
Self-concept	Characterization: event characteristic of oneself, how the event reflects personality, objectives, desires (self-reference)
Thinking	Internal: frequency of mental repetition
Conversation	External: frequency of external repetition, with friends and family for instance
Anticipated details	Prediction of details still present in a month, precision of the event in memory in a month of time given the number of details available
Reliving	To what extent the event is relived with details
Mental images	Number of mental images prompted by this event

The variables were subjectively assessed by the subjects on scales from 0 to 5, at the end of the encoding day, and at each retrieval.

#### 2.2.2 First recall

Four to 5 days after the encoding phase, participants had to give back all the material to the laboratory (notebook and wearable camera). They took part in a first surprise memory task (free recall). They were asked to recall aloud all the memories of the 20 events they had previously recorded with as many details as possible, such as the time and the location of the events, their perception and feelings of the events. At the end of the first recall, the participants assessed again the recalled events with the same subjective scales used at encoding (evaluation at the retrieval time). No feedback was given regarding the events that were not recalled.

#### 2.2.3 Second recall

**Figure 2:**
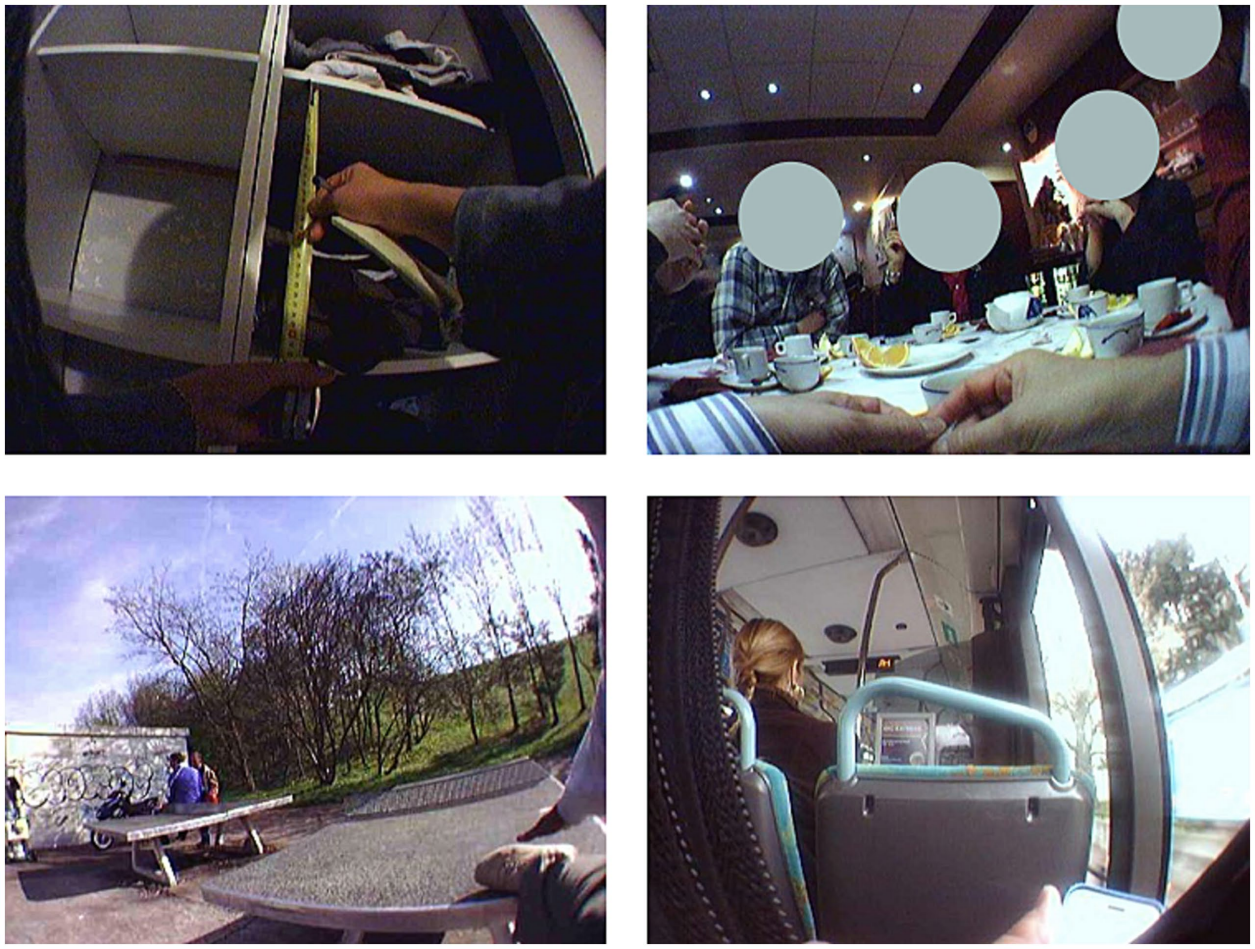
Examples of pictures taken by the participants using SenseCam.

One month after the end of the encoding phase, participants came back to the lab under the pretense of answering some final questionnaires. They took part in a second surprise memory task. This time, the free recall was followed by a cued recall for the events that were not recalled. Participants’ own SenseCam recordings were used as cues. Pictures of the general location where the events had taken place, with some specific details regarding the event and the people involved and taking part in the action, were presented (Figure 2). All the pictures were taken from a first-person perspective so that the participants might have seen parts of their bodies in the images. The pictures had to be well-lit and clear (e.g., a picture of a table at a restaurant). After the last recall, the participants assessed the events again with the same subjective scales (evaluation at the retrieval time).

#### 2.2.4 Objective scoring of the episodic nature of the memories

The researchers used a standard scale of EAM at the first and second recall to rate the episodicity of memories which takes into account the specificity of the event: unicity, spatio-temporal context, and richness of details [adapted from the TEMPau task, Piolino et al. (2006); for a review see Piolino et al. (2009)]. The 7-point scoring grid assessed different elements: the unicity of the event (“what”), the temporal context of the event (“when”: general time, with the duration and broad dating, and more precise temporal details, with a sequential localization in time), its spatial context (“where”: general localization context, then more detailed position in the setting), contextual non-temporal and non-spatial details (who was there, how did the event happen, diverse anecdotes…), and phenomenological context (thoughts, emotions, perceptions). The main details of each event could be controlled and checked using the SenseCam recordings. Each event was scored on a maximum of 7 points (global EAM score). Events were further differentiated in three categories based on their global EAM score: scores from 4 to 7 classified them as specific (EPI), a score of 0 classified the event as not recalled (NR). Memories scored from 1 to 3 were considered too vague and generic to be classified as specific and were therefore classified as semantic (SEM). Because there were not enough events in this category, it was not used in the analysis.

### 2.3 Data analyses

The memories constituted the observations. As some participants did not record every event as the instructions required, the total dataset comprised 414 observations. We used two dependent variables in separate analyses. The first set of dependent variables was the global EAM score and subjective assessments; the second was the events’ memory status (i.e., NR, EPI). Missing data in the subjective assessments was replaced using a principal component analysis (PCA) to preserve the trends in the dataset.

To test how performance and assessments would evolve with time, we first conducted a series of analyses of variance (ANCOVAs) on all the measures, with the time of the session as the within-subject factor while keeping the age of the participants constant. To determine the direction of the differences in the interactions between phases, we ran out post-hoc Bonferroni-corrected tests. The effect sizes were reported with partial eta squared (*η*^2^). In line with Guéguen (2009), we considered effect sizes as small for *η*^2^ < 0.06, medium for 0.06 < *η*^2^ < 0.14 and large for *η*^2^ ≥ 0.14.

The machine learning (ML) algorithm took all encoding and first recall variables, the age and the global EAM score for the first free recall, to predict the category of the second free recall (episodic or EPI versus not recalled or NR). Given that this accounts for a considerable number of variables, and to avoid any risk of overfitting, they were clustered using a principal component analysis (PCA), and only the corresponding “mean variable” of each cluster was considered in the training and testing of the classifiers. The PCA provided a total of 5 stable clusters (Table 2). The dataset was then randomly split into two parts: the training set, which would allow the ML algorithms to learn the patterns in the data to make their predictions, and the validation or test set, which would enable checking how well the ML algorithms worked. 80% of the dataset went into the training set, and 20% into the validation set. This partition was done randomly but preserved the repartition of the classes (EPI and NR) in each set. The algorithms that were tested were linear discriminant analysis, classification and regression trees, k-nearest neighbors, and random forest. The exact performance depended on the algorithm, but the conclusions presented here remain the same regardless of the nature of the classifier.

**Table 2:** Clusters for the ML algorithm as extracted with a PCA.

Cluster 1	Self-relevance (Encoding & Delay 1) Emotional intensity (Encoding & Delay 1) Conversation (Encoding & Delay 1) Thinking (Encoding & Delay 1)
Cluster 2	Emotional valence (Encoding & Delay 1)
Cluster 3	Remembering (Encoding & Delay 1) Perspective (Encoding & Delay 1) Self-concept (Encoding & Delay 1)
Cluster 4	Anticipated details (Encoding & Delay 1) Reliving (Encoding & Delay 1) Mental images (Encoding & Delay 1)
Cluster 5	Age Free recall at Delay 1

We used STATISTICA 12 (StatSoft, I, 2014) for the ANCOVAs and R (R-Core-Team, 2019) with the caret (Kuhn, 2008) and clustofvar (Chavent et al., 2012) packages for the rest of the analyses.

## 3 Results

After the one-month free recall, 144 of the 414 memories were recalled as episodic, that is, 34.8% of the events. 114 events were recalled only after the presentation of the visual cue. This number accounts for about a third (33.6%) of the episodic memories after the cued recall. In total, 339 events were classified as episodic memories for the cued recall, that is, 81.9% of the events. Table 3 summarizes the main results regarding the categories of the memories for the two free recalls. All the raw data are presented in Table 4 where the results of within-factor analyses are reported, controlling for the age of the participants. Overall, subjects had better memory scores (global EAM score) during the first free recall session (M = 3.64, SD = 2.26) than during the second one at a month (M = 3.02, SD = 2.37; *r* = 0.67, *p* < 0.05). However, when controlling for their age, the decrement was not significant anymore [*F*(1,412) = 0.22, *p* = 0.64]. Cued recall significantly improved the EAM score at 1 month [*F*(1,413) = 57.84, *p* < 0.001] and became a trend when controlling for the participants’ age [F(1,412) = 3.13, *p* = 0.077]. By contrast, most of the different scales used throughout the study were significantly impacted by time, independently of the age of the participants. Repeated measures ANCOVAs showed a significant effect of time (*p* < 0.05) for remembering, perspective, self-concept, thinking, conversation, anticipated details, reliving, and mental images. The scores declined from encoding to second recall regarding remembering, perspective, self-concept, thinking, reliving and mental images (vividness). Scores concerning the frequency of rehearsal through conversation were significantly inferior at the second recall compared to those of the encoding and first recall. Finally, scores of anticipated details at the one-month retrieval were higher at encoding than the first and second recalls. There was no time effect regarding emotion intensity, emotion valence, and self-relevance.

**Table 3:** Summary of the main results for the two recall phases.

First free recall	Second free recall – category after first recall	Second free recall – total	Cued recall – category after second free recall	Cued recall – total
Episodic (182)	Episodic: 136	*Episodic: 144*	Episodic: 143	Episodic: 339
	Generic: 9		Generic: 0	
	Not recalled: 37		Not recalled: 1*	
Generic (131)	Episodic: 5	Generic: 125	Episodic: 114	Generic: 43
	Generic: 107		Generic: 11	
	Not recalled: 19		Not recalled: 0	
Not recalled (101)	Episodic: 3	*Not recalled: 145*	Episodic: 82	Total: 32
	Generic: 9		Generic: 32	
	Not recalled: 89		Not recalled: 31	

The number of episodic memories (EPI), generic memories (recalled, but without enough details to be episodic), and not recalled memories for each recall is provided for the first free recall. The evolution of the status of the memories from the first free recall (5 days delay) to the second free recall is indicated in the ‘Second free recall – category after first recall’. For instance, among the 182 episodic memories at the first free recall, 136 were episodic at the second free recall. The ‘Second free recall – total’ indicates the total for each category at the one-month free recall (sum of the different items in the ‘Second free recall – category after first recall’). Likewise, the last two columns indicate the category of the cued recall events after the second free recall and their respective totals. The events used for the ML analyses are in italics (*only one event, for one participant aged 22, was not recognized based on the pictures after being freely recalled as episodic).

**Table 4:** EAM and characteristics of memories from encoding to delayed retrieval and results of ANCOVAs controlling for the age of the participants.

Mean scores	Encoding	First recall	Second recall	F
EAM (free recall) [cued recall]		3.64 (2.25)	3.02 (2.36) [4.45 (1.62)]	F(1,412) = 0.22 *η*^2^ = 0.00
Emotion (intensity)	2.52 (1.38)	2.60 (1.15)	2.46 (1.28)	*F*(2,824) = 1.65 *η*^2^ = 0.00
Emotion (positive valence)	3.09 (1.27)	2.95 (1.12)	2.86 (1.19)	F(2,824) = 0.83 *η*^2^ = 0.00
Self-relevance	2.38 (1.43)	2.34 (1.27)	2.28 (1.41)	F(2,824) = 2.33 *η*^2^ = 0.00
Remembering	3.27 (1.44)	2.81 (1.40)	2.37 (1.46)	F(2,824) = 14.83***1 *η*^2^ = 0.03
Perspective (1PP)	3.59 (1.49)	3.19 (1.35)	2.74 (1.64)	F(2,824) = 21.27***1 *η*^2^ = 0.05
Self-concept (defining)	2.72 (1.43)	2.51 (1.27)	2.17 (1.37)	F(2,824) = 8.46***1 *η*^2^ = 0.02
Internal rehearsal (thinking)	1.89 (1.60)	1.66 (1.36)	1.27 (1.32)	F(2,824) = 5.03**1 *η*^2^ = 0.01
External rehearsal (conversation)	1.31 (1.48)	1.23 (1.31)	1.07 (1.19)	F(2,824) = 3.11*2 *η*^2^ = 0.00
Anticipated details	2.04 (1.53)	1.69 (1.34)	1.57 (1.33)	F(2,824) = 3.84*3 *η*^2^ = 0.01
Reliving details	2.24 (1.56)	1.95 (1.37)	1.74 (1.40)	F(2,824) = 3.41*1 *η*^2^ = 0.00
Vividness (mental images)	2.56 (1.52)	2.21 (1.36)	1.97 (1.36)	F(2,824) = 3.12*1 *η*^2^ = 0.00

Bonferroni *post-hoc* tests: 1: each pairwise comparison is significant; 2: Delay 2 is significantly inferior to Encoding and Delay 1; 3: Encoding is significantly superior to Delay 1 & 2.

**Figure 3:**
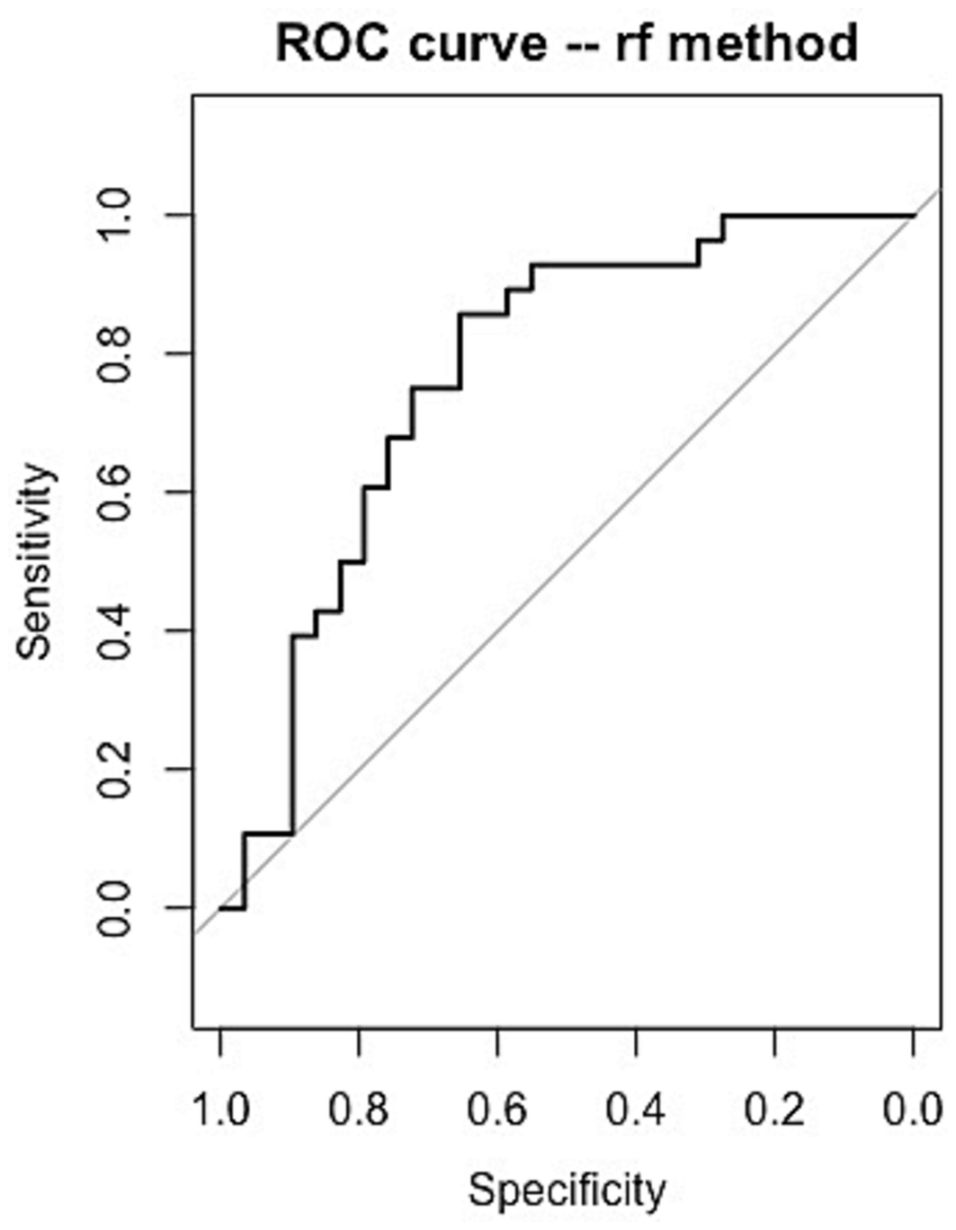
ROC curve for the prediction in Table 7. AUC = 0.772.

Exploratory analyses based on ML were carried out to determine whether the memory status at a month could be predicted. The main goal was to assess how well a trained classifier algorithm could predict the nature of an event in memory at the long-term free recall (either well remembered, EPI, or not remembered, NR, see Methods: 144 episodic memories EPI at the one-month free recall versus 145 not recalled NR, for a total of 289 events). It was not possible to train classifiers on the cued recall as the difference between the numbers of episodic memories (339) and not recalled events (32) was too high. Overall, for the one-month free recall, the accuracy fell around 75% as a mean for all ML algorithms, and kappa around 0.50. The best algorithm was random forest (rf), with a classification’s mean accuracy of 78% (Table 5) and a Cohen’s kappa value of 0.56 (Table 6). The accuracy is satisfactory as the chance level here is about 50%. The kappa value, measuring the inter-trial reliability, can be considered a fair one, which is confirmed by the confusion matrix of the predictions on the validation dataset (Table 7). On the validation or test dataset, the model correctly predicted 25 of 28 episodic memories, and 17 of 29 not-recalled events, reaching a correct accuracy for both categories. The receiver operating characteristic (ROC) curve (Figure 3) showed an area under the curve (AUC) of 0.772. Table 8 gives the respective weights of the clusters in the model: the best predictors among the phenomenal variables concerned anticipated long-term details, reliving details, mental images, and the self-reference at encoding and at the first free recall.

**Table 5:** Accuracy of the ML model on the training set for the best algorithm (rf).

Model	Min.	1st Qu.	Median	Mean	3rd Qu.	Max.
rf	0.652	0.742	0.771	0.781	0.824	0.875

**Table 6:** Cohen’s kappa values of the ML model on the training set for the best algorithm (rf).

Model	Min.	1st Qu.	Median	Mean	3rd Qu.	Max.
rf	0.308	0.489	0.542	0.562	0.652	0.750

**Table 7:** Confusion matrix of the prediction on the testing set of the ML model for the best algorithm (rf).

	EPI	NR
EPI	25	3
NR	12	17

The validation dataset corresponds to the rows and the predictions to the columns. 25 “EPI” memories were correctly classified and three were misclassified; 17 “NR” memories were correctly classified and twelve were misclassified.

**Table 8:** Scaled importance of the clusters in the final model.

	Raw importance	Scaled importance
Cluster 5	54.06	100.000
Cluster 4	17.67	11.763
Cluster 3	15.72	7.039
Cluster 1	15.25	5.891
Cluster 2	12.82	0.000

## 4 Discussion

In daily life, the encoding of unique events relies on variables related to the idiosyncratic values of these events for a person. Determining whether the subsequent episodic memories are predictable, depending on the subjective variables at encoding, some being better predictors than others, could become an asset in memory research and therapy. The main aim of this study was to assess the predictability of real-life episodic memories (i.e., episodic autobiographical memories) based on a given dataset acquired using SenseCam technology. Different subjective characteristics of events were recorded during the encoding phase and two subsequent recalls to provide potential inputs to predict the final mnesic state of the—remembered or not—events at a delay of 1 month. The memory scores tended to decline depending on how much time had passed since encoding. Some phenomenological variables proved to be excellent predictors, such as the mental images, the reliving of details, the Self and the amount of anticipated details in memory at encoding and first recall; moreover, the age of the subjects had a strong influence on the dataset, which points to a role of age in highly ecological memories. Likewise, the memory performance at first recall impacted the prediction of the one-month recall, revealing the role of the consolidation process in this experiment. The final exploratory classifier model (random forest) had a 78% mean accuracy, providing some evidence that such predictions based mainly on subjective assessment could be possible.

In the present study, the decline of the memory score over the one-month time interval depended on the age of the participants. The influence of age is consistent with previous findings concerning EAM [see notably Faßbender et al. (2022), Mair et al. (2021), Sander et al. (2021), Armson et al. (2017), Martinelli et al. (2013a), Piolino et al. (2006)] as well as for the memory of new naturalistic events (Plancher et al., 2010; Abichou et al., 2019). Nevertheless, independently of the participants’ age, we found a decline of the subjective self-rated scales over time (e.g., remembering, first-person perspective, reliving details, vividness), which is consistent with previous literature, in particular regarding the recency effect of the EAM retention curve as first described by Rubin (1982) and further experimentally confirmed by Piolino et al. (2002) for instance. In previous SenseCam studies, Finley et al. (2011) also found a decreased picture-cued recall over time. By contrast, the emotion and the self-relevance remained stable over time and were limited predictors of long-term recall compared to other phenomenological measures. In general, item-emotion binding has a well-established role in memory encoding and consolidation [see for instance Allen et al. (2008), Sheldon et al. (2020), Talmi et al. (2019), Yonelinas and Ritchey (2015)], and so does self-relevance (Straube, 2012; Penaud et al., 2022). A possible explanation for this result lies in the very nature of the events recorded via SenseCam by the participants. The events were indeed part of their daily lives, and they thus might not have been emotional and salient enough to allow a more discriminant consolidation role of these variables from encoding to predict long-term retrieval.

The first recall memory score and the subjective assessments regarding anticipated long-term memory details, the reliving of details, and the mental images at both encoding and the first recall were the best predictors of long-term recall, proving their fundamental importance in building long-lasting EAM. This finding is in keeping with previous literature that subsequent recalls or updates of information (Alberini, 2005; Dudai, 2012) are important determinants of whether some information will be captured in long-term EM or forgotten. Moreover, it is noteworthy that mental images have a substantial role in the properties of EM (recollection of events with spatial–temporal context and perceptual details, see Tulving (1985)). In the same line, visual mental imagery provides rich contextual, sensorial and perceptual information that enhances access to specific autobiographical memories (Conway and Pleydell-Pearce, 2000; Aydin, 2018) and EM (Hussey et al., 2012; Barry et al., 2019) and the sense of Self (Lin, 2018). With the importance of the anticipated long-term details at encoding for long-term recall, the subjects also seemed to anticipate how the experienced event would be later remembered. This is a direct translation of the prospective brain and the ability of EM to project into the future (Buckner, 2007; La Corte et al., 2021). Prospection and future-oriented cognition describe the capacity to envision the possible future which encompasses episodic simulation, prediction, intention, and planning. The present result extends the importance of episodic prediction, which concerns the estimation of the likelihood of a specific episodic autobiographical future event, to the estimation of the nature of subsequent long-term EM. Other factors about metacognition assessments also play a role in this prediction, notably belief, which is distinct from recollection (Fitzgerald and Broadbridge, 2013), with a double dissociation including different factors predicting autobiographical belief and autobiographical recollection (Scoboria et al., 2014).

Other variables from the subjective assessments stood out as decent predictors, though less important than the previous ones, and seemed to point at the idiosyncratic criteria of selection in daily life by the subjects and at self-reference processes. Besides the role of actor-perspective and the sense of remembering via autonoetic consciousness at encoding and first recall, which are closely related to mental imagery and the reliving of details and the first-person perspective, the implication of the self-concept in predicting long-term recall highlighted the role of the Self in long-lasting EM or EAM (Conway and Pleydell-Pearce, 2000; Conway, 2005; Piolino et al., 2009; Staniloiu et al., 2020). In particular, Conway introduced the notion of *working self* which corresponds to a complex, dynamic and executive set that contains active goals and personal aspirations in the short and long term. Its central role is to maintain coherence between goals and memories. The link with the Self is a constant in long-term, autobiographical-like memories, as the core of the self-memory system (Conway and Pleydell-Pearce, 2000; Martinelli et al., 2013b). For 40 years, many experimental studies have shown the role of the Self in EM encoding using the manipulation of self-reference (relating a new piece of information in some way to oneself) in a laboratory context (Symons and Johnson, 1997; Klein, 2012) as an experimental simulation of the formation of EAM (Lalanne et al., 2013). In the present study using SenseCam, which can be considered as an ecological self-referential paradigm, the participants had to decide by themselves to record personal moments, even the most mundane ones, which were, thus, more or less explicitly linked to their self-images and current self-concepts (goals, values, desires…). Moreover, each recorded event involved the Self as an agent (‘I’) that was reexperiencing previous real-life personal events in a first-person perspective, a prerequisite for rich EM (Prebble et al., 2013; Bergouignan et al., 2014; Iriye and St. Jacques, 2021). The subjective assessment of the “perspective” element (actor vs. observer) was linked with the free recall score, in line with these elements. A recent study by Baldwin et al. (2021) investigated the effect on memory of the self-choice, that is, freely chosen events to remember versus assigned ones: self-choice improves memory, but prior actions reflecting self-control (for instance, consuming self-regulatory resources with a Stroop task), that is, ego depletion, reduce this influence. Interestingly, self-relevance was a limited predictor compared to the connection of events with self-concept, which may indicate a difference between those two notions related to the Self (Brechet et al., 2020; Morin and Racy, 2021; Tanguay et al., 2022). Another explanation could be that the events were not fully self-defining memories, as attested by the effect of the age found in our sample, which generally disappears in the case of memories highly related to the Self (Martinelli et al., 2013a).

Finally, event memory rehearsal at encoding and first recall appeared as a very limited predictor. Memory repetition, that is, thinking about the event and talking about it with others, is well known to be linked with the adaptative process of (re)consolidation (Nadel and Moscovitch, 1997; McGaugh, 2000; Wichert et al., 2011; Dudai, 2012; Squire et al., 2015; van Ekert et al., 2017; Yonelinas et al., 2019; Cowan et al., 2021); this link has notably been explored in relationship with mind-wandering (Girardeau et al., 2023). However, in our results, both the internal and external repetition were among the least strong predictors of the free recall memory score at a month. These predictors appeared in the same cluster as emotional intensity and self-relevance, hinting at the links between these measures for long-term EM [see, for instance, D'Argembeau et al. (2005), Kalenzaga et al. (2013), Durbin et al. (2017), Moses-Payne et al. (2022)]. With longer delays, internal and external repetitions could be more decisive in predicting long-term EM or its generalization or semanticization (Cermak, 1984).

To our knowledge, no other study using SenseCam tested different recall times with a focus on different subjective factors characterizing real-life events. The present study thus provides a first approach in this field, with a promising prediction of potential long-term memory episodicity of encoded events in ML. This naturalistic approach leads to consider long-lasting EM with its encoding variables in the most ecological setting possible. Indeed, using SenseCam allowed an experimental paradigm that was as ecological as possible in a real-life situation, as the participants kept on doing their daily activities, and freely chose the events they recorded. The naturalistic, ecological aspect of SenseCam paradigms has been checked by studies indicating similar neural activation as during EAM retrieval [see for instance Milton et al. (2011), Cabeza et al. (2004)]. Nonetheless, this study presents some limitations that should be considered in future studies. First, we addressed mundane real-life events and a long-term delay recall of a month. The predictors could differ with more remarkable events and/or longer delays. Second, distinct age cohorts would help disentangle the exact role of age in this context. Besides, adding other experimental groups with only one recall time would provide a comparison controlling for forced re-consolidation through testing. The further investigation of a longer delay for the cued recall could lead to decreased performance for the participants (Tulving et al., 1982), and thus allow the comparison of classifiers trained on the free recall, versus trained on the cued recall. All things considered, this dataset was best described by measures related to mental imagery (e.g., number of images, reliving details), self-reference (self-concept, autonoetic consciousness, first-person perspective), and prospection (anticipated details). The critical role of these factors for EAM consolidation suggest their potential role in enhancing the construction phase of retrieval over time, as regions associated with mental imagery, self-reference, integration of perceptual details, multisensory features, and social processes are selectively activated during this early phase (Daviddi et al., 2023). Investigating this finding using fMRI during the early and late phases of EAM retrieval at different retention delays from encoding would shed further light on this issue. Furthermore, it would be quite interesting to test the same algorithms with different datasets or, better yet, with datasets testing the ecological validity of the encoding environment, that is, comparing SenseCam with other ecological paradigms, such as virtual reality. Such a naturalistic approach would increase our understanding of the formation of autobiographical memory and the joint influences of the encoding variables on later recollection. From a clinical perspective, the results of this study suggest that instructing patients to pay particular attention to certain features of their real-life events as they occur (taking ‘mental pictures’, reflecting on how they relate to themselves, what they might remember in the future…) and then rehearsing them both mentally and verbally with other people could potentially lead to better memory performance. Rehabilitation and training using SenseCam usually focus on repeated review of recorded events to strengthen consolidation and retrieval, rather than better encoding (Woodberry et al., 2015; Mair et al., 2019). EAM rehabilitation could thus benefit from taking into account both the influence of the encoding and retrieval variables, to design protocols aiming at maximum efficiency with adapted procedures, and the importance of an ecological design to apply the protocols more easily to real-life situations (Dubourg et al., 2016; Silva et al., 2018).

## Data availability statement

The raw data supporting the conclusions of this article will be made available by the authors, without undue reservation.

## Ethics statement

The studies involving humans were approved by the Paris Institute of Psychology local committee. The studies were conducted in accordance with the local legislation and institutional requirements. The participants provided their written informed consent to participate in this study.

## Author contributions

DL: Data curation, Formal analysis, Software, Visualization, Writing – original draft, Writing – review & editing. BF: Formal analysis, Software, Writing – review & editing. PP: Conceptualization, Data curation, Formal analysis, Investigation, Methodology, Supervision, Writing – review & editing.
